# Key Signalling Molecules in Aging and Neurodegeneration

**DOI:** 10.3390/cells11050834

**Published:** 2022-02-28

**Authors:** Riccardo Filadi, Paola Pizzo

**Affiliations:** 1 Department of Biomedical Sciences, University of Padua, 35131 Padua, Italy; riccardo.filadi@cnr.it; 2 Neuroscience Institute, National Research Council (CNR), 35131 Padua, Italy

One of the major challenges of modern medicine is to block or prevent the neurodegenerative processes inevitably associated with different pathological conditions. The number of cases of neurodegenerative disorders such as Parkinson’s (PD) and Alzheimer’s disease (AD) has dramatically increased in recent decades, in parallel to an increase of the human lifespan. While aging represents a key risk factor for the onset of these disorders, the failure of most of the clinical trials and the lack of effective therapeutic approaches suggest the urgent need to deepen the early mechanisms underlying neurodegeneration. Therefore, this Special Issue of *Cells* focuses on the study of key signalling pathways and molecules whose alterations have been associated with a specific neurodegenerative condition, or suggested to be conserved in different pathologies.

Among these molecules, Sonika Rathi et al. [1] investigated the role of the CDKN2B-AS1 and CDKN2B genes in the 9p21 locus in primary open-angle glaucoma, concluding that the former gene might act as a regulator modulating the expression of the second one. Veronika Matschke and co-workers found a tissue-dependent regulation of the microRNA *miR*-*29b*-*3p* expression in the cerebellum and cervical spinal cord of wobbler mice (a model of amyotrophic lateral sclerosis, ALS), suggesting that a tissue-specific regulation of the intrinsic apoptotic pathway might be involved in the disease [2].

The paper by Mathilde Chivet et al. highlighted how, in different mouse models, polyglutamine (polyQ) expansions in the androgen receptor (AR) gene, associated with loss of lower motor neurons in spinal and bulbar muscular atrophy (SBMA), causes AR nuclear enrichment and aggregation in myofibers, a process (enhanced by sex and age) triggering muscle atrophy and premature death [3]. The importance of the fine characterization of specific mouse models of disease was also suggested by the paper of Francesca De Giorgi, François Ichas et al., whereby it was demonstrated that the targeted overexpression of human α–synuclein (α-syn) in oligodendrocytes does not recapitulate the appearance of α-syn fibrillary aggregates (observed in the brains of patients affected by multiple system atrophy, MSA), but it can work as the basis for seeding aggregation upon exposure to α-syn preformed fibrils [4].

Maria Cano-Abad and co-workers identified the Ca^2+^ channel Calcium homeostasis modulator 1 (CALHM1) as a target for neuroprotection against ischemia [5], whereas Jong Heo et al. suggested that the potentiation of the Stromal cell-derived factor 1 (SDF-1)-CXC chemokine receptor 4 (CXCR4) axis might protect vascular endothelial cells of the brain from damage associated with ionizing radiation treatment [6]. Notably, the activation of the CXCR4 signaling pathway, either by a natural ligand (CXCL12α) of this receptor or by the small molecule NUCC-390 (a CXCR4 agonist), has been demonstrated by Samuele Negro et al. to promote nerve recovery at the neuromuscular junction upon α–Latrotoxin-induced degeneration of motor axon terminals [7]. The existence of endogenous signals mediating neuronal self-repair and/or protection from pathological stimuli, both in the peripheral [8] and central nervous system [9], has been the topic of two interesting reviews by Michela Rigoni and David Romeo-Guitart, and respective collaborators, suggesting these pathways could be promising pharmacological targets to reduce and/or revert the neurodegenerative process.

Among the routes frequently associated with neurodegeneration and neuronal repair in different pathological conditions, a special role is that exerted by the signaling cascades involving: (a) Epidermal growth factor receptor (EGFR), which has been suggested to be endowed with neurotrophic functions, as described by Roberta Romano and Cecilia Bucci [10]; (b) G-Protein-Coupled Receptors, presented by Choi Dong-Kug and collaborators as key targets of several pharmacological approaches under development [11]; (c) compartmentalization of cyclic AMP (cAMP) signals, reviewed by Di Benedetto et al. [12]; (d) mitochondrial metabolism. Dysregulation of this latter is a typical trait observed in different neurodegenerative and aging-related disorders, ranging from PD (where it is associated with alterations of neuronal Ca^2+^ homeostasis, as described by Enrico Zampese and James Surmeier [13]), to AD (see below) and aging-related sarcopenia, as reported by Vanina Romanello et al. [14].

AD is the most frequent age-related neurodegenerative disease in the world. In this Special Issue, numerous papers focused on this disease, highlighting multiple pathogenic processes and key signaling molecules, here suggested as new potential targets for therapeutic interventions aimed at contrasting the neurodegenerative process. Firstly, in the work of Alejo Nevado-Holgado et al. [15], the JAK-STAT signaling axis is indicated as a key pathogenic pathway in AD. By a promising multi-modal approach that combines genome-wide association studies (GWAS) with clinical data and RNA expression analyses from AD patients, the authors found altered regulation of JAK-STAT pathway-related genes in the pathology, an observation further validated by in vitro and in vivo experiments in AD models.

Carmela Matrone and collaborators [16], instead, underlined the importance of the Fyn tyrosine kinase-dependent APP phosphorylation event in promoting amyloidogenic APP processing and neuronal death. Indeed, they showed that upon APP phosphorylation at Tyr682 by Fyn kinase, APP is forced into neuronal acidic compartments to generate toxic Aβ peptides. Interestingly, this pathway results in an increase in AD neurons, which also shows a potentiated Fyn activity, making APPTyr682 and Fyn kinase good candidates for diagnostic purposes and pharmacological targeting, respectively, in AD.

Other important molecules in AD pathogenesis are suggested by Evgeny Barykin et al. [17], who found that the Aβ modified peptide iso-Aβ, linked to neurotoxicity and amyloidosis, is a potent inhibitor of the nicotinic acetylcholine receptor (α7nAChR), by binding to it in allosteric mode. Interestingly, the authors showed that the iso-Aβ peptide has increased neurotoxicity, compared to that of the unmodified Aβ, which is linked to its higher efficacy in blocking the α7nAChR-mediated Ca^2+^ current, an important event in the disruption of the cholinergic system in AD.

A key role for the stem cell-related pluripotency factor Nanog in AD is also proposed in the paper of Ching-Chi Chang et al. [18]: its upregulation reduced Aβ toxicity and cell senescence in vitro and improved memory and learning in a rat model of Aβ-induced neurotoxicity, suggesting that its pharmacological/genetic manipulation could represent a strategy for blunting Aβ toxicity in AD brain.

Neuroinflammation, a pathogenic process associated with AD, as well as with the majority of neurodegenerative diseases, is analyzed by Cinzia Severini and collaborators [19]. By using different AD animal models, as well as samples from AD patients, the authors found, compared to controls, increased mRNA and protein levels of the inflammatory chemokine prokineticin 2 (PROK2), both in the brain and in the serum, identifying a potential new biomarker of the disease.

In the review of Shang-Der Chen et al. [20], the function of the helix-loop-helix proteins of the Id (Inhibitor of DNA-binding/differentiation) family is deeply analyzed. These proteins are critical for regulating, among other processes, cell cycle re-entry and may play a central role in Aβ-induced neuronal demise. Multiple mechanisms proposed for cell cycle dysregulation in AD and recent evidence on the function of Id proteins in regulating neuronal fate are presented, suggesting early interventions for AD to put in action before apoptosis of fully differentiated neurons during the progression of the pathology.

Maria Ankarcrona et al. [21] deals with an emerging aspect in neurodegeneration which is the altered communication between the endoplasmic reticulum (ER) and mitochondria. The authors reported an increased juxtaposition between ER and mitochondria in the brain of different AD mouse models, in primary neurons from these mice, as well as in wild-type neurons treated with Aβ. Importantly, the potentiated inter-organelle tethering found in these cells is linked to dysfunctions in autophagy and mitochondrial bioenergetics.

Ultimately, three different papers analyzed the role played by presenilin 2 (PS2), one of the three mutated proteins causing familial AD (FAD), in determining, by multiple means, neuronal dysfunction and degeneration. Firstly, Giulia Rigotto et al. [22] investigated possible mitochondrial alterations in neurons from FAD-PS2 mice, as a key mechanism for neurodegeneration. They found defects in mitochondrial respiration, organelle membrane potential and Ca^2+^ handling in AD neurons under mild stress that were partially rescued by an inhibitor of the mitochondrial permeability transition pore, proposing the latter as a possible tuneable target to potentiate mitochondrial and cellular function in AD. Alice Rossi et al. [23] investigated the role of endogenous PS2 in neuronal function, by using knock-out (KO) animals for the protein. They showed that PS2-KO primary cortical neurons present a decreased ER-mitochondria coupling and a blunted mitochondrial Ca^2+^ signal upon neuronal stimulation, as well as a slight alteration in organelle respiration, confirming the key role played by PS2 in keeping mitochondrial health. Moreover, the paper by Michela Rossini et al. [24] identified the domain of PS2 which is involved in the previously described capacity of the protein to reinforce ER-mitochondria coupling. Specifically, the sole cytosolic loop of PS2, when targeted to the mitochondrial surface, was able to counteract the activity of FAD-PS2 mutants on organelle tethering, proving itself as a possible tool for recovering FAD-PS2-associated alterations linked to this signaling axis.

Last, but not least, two interesting reviews present different tools and animal models available for studying Ca^2+^ signaling in neuronal physiology and during aging and neurodegeneration. The first, by Nelly Redolfi et al. [25], is an overview of the most used model organisms employed for Ca^2+^ imaging in brain research, from *D. melanogaster* to *C. elegans*, from different transgenic mouse models to zebrafish lines, underlining their specific applications in the field. The review by Javier Alvarez, Mayte Montero and collaborators [26], instead, focuses only on *C. elegans*, presenting all the advantages in using the nematode as a model organism to understand in vivo the role of Ca^2+^ signaling in aging and neurodegeneration, being transparent throughout its life, and thus suitable for the expression of Ca^2+^ fluorescent probes, and presenting gene mutations linked to different neurodegenerative diseases.

In addition to the importance of better defining the early events leading to neurodegeneration, a key step in the effort of preventing the onset of blocking the progression of neurodegenerative disorders is the development of methods and tools for an early and precise diagnosis. Indeed, one of the reasons underlying the lack of success of several clinical trials is likely a tardive patient recruitment, i.e., when the symptoms are evident and the degenerative process is already irremediable. In this context, the use of free-water imaging to detect and distinguish neuronal degeneration and neuroinflammation in the white and grey matter of PD patients, as shown by Koji Kamagata and co-workers [27], appears promising. Similarly, in a FAD mouse model, the finding of early (i.e., before amyloid deposition), specific patterns of alterations in brain electrical activity suggests the possibility to use this tool for an early AD diagnosis, as presented by Cristina Fasolato and collaborators [28]. Moreover, in the paper by Lu Zhao et al. [29], the introduction of novel tools to investigate in vitro and in vivo the activation of specific cell pathways, such as the pHluorin-BACE1-mCherry reporter for BACE1 distribution and activity, appears of utmost importance to screen possible therapeutic candidates.

In conclusion, this Special Issue provides new molecular advice to sketch a better scenario for understanding aging processes and neurodegenerative events, stimulating further research for the development of early and efficient therapeutic interventions for related pathologies.
